# Increasing the availability of health workers in rural sub-Saharan Africa: a scoping review of rural pipeline programmes

**DOI:** 10.1186/s12960-023-00801-z

**Published:** 2023-03-14

**Authors:** Delphin Kolié, Remco Van De Pas, Laurence Codjia, Pascal Zurn

**Affiliations:** 1 Maferinyah National Training and Research Centre in Rural Health, Ministry of Health, Forecariah, Guinea; 2 Department of Public Health, Institute of Tropical Medicine of Antwerp, Antwerp, Belgium; 3 Department of Health Workforce, World Health Organisation, Geneva, Switzerland

**Keywords:** Rural pipeline programmes, Medical education reforms, Health workers, Sub-Saharan Africa, Scoping review

## Abstract

**Introduction:**

Rural pipeline approach has recently gain prominent recognition in improving the availability of health workers in hard-to-reach areas such as rural and poor regions. Understanding implications for its successful implementation is important to guide health policy and decision-makers in Sub-Saharan Africa. This review aims to synthesize the evidence on rural pipeline implementation and impacts in sub-Saharan Africa.

**Methods:**

We conducted a scoping review using Joanna Briggs Institute guidebook. We searched in PubMed and Google scholar databases and the grey literature. We conducted a thematic analysis to assess the studies. Data were reported following the PRISMA extension for Scoping reviews guidelines.

**Results:**

Of the 443 references identified through database searching, 22 met the inclusion criteria. Rural pipeline pillars that generated impacts included ensuring that more rural students are selected into programmes; developing a curriculum oriented towards rural health and rural exposure during training; curriculum oriented to rural health delivery; and ensuring retention of health workers in rural areas through educational and professional support. These impacts varied from one pillar to another and included: increased in number of rural health practitioners; reduction in communication barriers between healthcare providers and community members; changes in household economic and social circumstances especially for students from poor family; improvement of health services quality; improved health education and promotion within rural communities; and motivation of community members to enrol their children in school. However, implementation of rural pipeline resulted in some unintended impacts such as perceived workload increased by trainee’s supervisors; increased job absenteeism among senior health providers; patients’ discomfort of being attended by students; perceived poor quality care provided by students which influenced health facilities attendance. Facilitating factors of rural pipeline implementation included: availability of learning infrastructures in rural areas; ensuring students’ accommodation and safety; setting no age restriction for students applying for rural medical schools; and appropriate academic capacity-building programmes for medical students. Implementation challenges included poor preparation of rural health training schools’ candidates; tuition fees payment; limited access to rural health facilities for students training; inadequate living and working conditions; and perceived discrimination of rural health workers.

**Conclusion:**

This review advocates for combined implementation of rural pipeline pillars, taking into account the specificity of country context. Policy and decision-makers in sub-Saharan Africa should extend rural training programmes to involve nurses, midwives and other allied health professionals. Decision-makers in sub-Saharan Africa should also commit more for improving rural living and working environments to facilitate the implementation of rural health workforce development programmes.

## Introduction

The global shortfall of health workers (HWs) was estimated at 15 million in 2020 [1]. A quarter of this need-based shortage is found in sub-Saharan Africa (SSA) region; sorely confronted with endemic health problems (Malaria, HIV/AIDS, TB, etc.) and recurrent outbreaks of re-emerging infectious diseases, such as Ebola, Covid-19 [2, 3]. The inequitable distribution of HWs found between SSA and other regions of the world is likewise reflected within SSA countries and rural, peri-urban and urban locations [4]. Nowadays, most SSA countries display serious imbalances of HWs distribution between rural and urban areas, such that half of rural population are denied essential healthcare services [5]. This situation hinders healthcare systems capabilities in SSA to achieve high and effective coverage of health services [6]. Moreover, as epidemiological transition becomes obvious, and demographic change irreversible in SSA, predictions sustain that healthcare demands and needs in this region will grow at an unprecedented pace in the nearby future [7, 8]. Hence, the need to improve the availability and employment of qualified and fit-for purpose HWs in SSA, especially in rural and remote locations, is more imperative now than ever [9].

In an effort to improve HWs development, attraction, recruitment, and retention in rural and remote areas, the World Health Organization (WHO) has recommended set of strategies including those pertaining to the development of HWs. Notably, targeted admission policies to enrol students with a rural background in HWs education programmes; locating health education facilities closer to rural areas; exposing students of a wide array of HWs disciplines to rural and remote communities and rural clinical practices; including rural health topics in HWs education. The WHO recommends a policy of having career development and advancement programmes, and career pathways for HWs in rural and remote areas [5]. Together, this comprehensive rural targeting and training in rural areas can be referred as a “rural pipeline” approach [10]. The rural pipeline approach has four tailored pillars: (i) advocating careers in health professions among rural students; (ii) prioritizing the selection of rural students into programmes; (iii) developing a curriculum oriented towards rural health and rural exposure during training and (iv) ensuring retention of HWs in rural areas through educational and professional support [10].

Rural pipeline programmes have yielded positive results in many High-and-Middle Income countries like the USA, Canada, Australia, Sweden, and Norway [10–19]. For instance, authors in the USA have documented the persisting impact of rural pipeline interventions in retaining up to 87% of programmes’ graduates in underserved areas 22 years after their recruitment [20]. In six Northern European countries, including Sweden and Norway, 67% of health professionals under rural pipeline programmes were retained in rural and underserved locations five years after their recruitment [21].

Rural pipeline implementation is relatively new to SSA settings. However, there exists well known documented experiences from countries like South Africa [22], Uganda [23, 24], and Mali [25, 26]. For instance, Dormael and colleagues in their study on recruiting medical doctors to rural districts found that preferential selection of students with rural background and their exposure to rurally relevant training packages enhanced their self-confidence, job-satisfaction and retention during the three years of the project lifespan in rural Mali [27]. Authors have also reported the positive outcomes of rural pipeline programmes on the attitudes, specialty choice, and intentions of HWs to practice in rural areas; and their performance in local health care systems in SSA [28–31]. Nonetheless, the successful implementation of these strategies are context-dependent. Factors that hinder the sustainability of these strategies include financial, structural and managerial challenges [32, 33].

In a recent systematic review, the WHO has reported on strategies that increase the retention of HWs in rural areas in the global context, including in SSA [5]. However, studies included in this review were unequally distributed across the WHO region, with only 18% from SSA [5]. Additionally, out of the 22 studies selected from SSA, only four were about medical education reform [5]. While this review acknowledged that successful implementation of these strategies are context-dependent, it did not, however, disaggregate findings by WHO region [5]. This raises the need for more in-depth insights of rural pipeline strategies implemented in SSA region, and their impacts.

We therefore undertook this scoping review of the literature relating to the status of rural pipeline programmes implementation and impacts in SSA. Specifically, we attempted to:

- To identify rural pipeline strategies/pillars implemented to improve the availability of qualified HWs in rural SSA and their impacts on health services and systems;
- To assess facilitators and challenges for the implementation of rural pipeline strategies to improve the availability of qualified HWs in rural SSA.

## Methods

### Study design

This scoping review uses the guidance document developed by the Joanna Briggs Institute (JBI) [34]. We opted for this study design because of the scope of our research questions, the potential of this methodological approach to map and summarize empirical evidence, and inform future research.

We followed the five stages for scoping review outlined by the JBI manual: (1) identification of research questions; (2) identification of relevant studies; (3) study selection; (4) data charting; and (5) results summarizing and reporting [34].

### Identification of research questions

For this scoping review, three key research questions were identified: (i) what is known on rural pipeline strategies or its pillars implemented to improve the availability of qualified HWs in rural Africa, (ii) what are the facilitators and challenges for the implementation of rural pipeline strategies to improve the availability of qualified HWs in rural SSA, and (iii) what have been the impacts of rural pipeline strategies on improving the availability of qualified HWs in rural SSA?

Through these questions, we sought to map and summarize the range of rural pipeline interventions undertaken in SSA to improve the attraction and retention of qualified HWs in rural and remote areas.

### Identification of relevant studies

We used PubMed and Google scholar databases to search for empirical peer-reviewed literature that reported on rural pipeline interventions for increasing the availability of qualified HWs in rural SSA. These studies were published in English or French. The peer-reviewed literature search strategy was based on the “PCC approach” (Population, Concept and Context) of the JBI guide and included studies from 2000 onwards:

- Population: This included the formal health workforce taking into account their varying profile (medical doctors or physician, nurses or nurses practitioners, midwives or nurse-midwives, etc.);
- Concept: search terms included “rural pipeline” or “rural pathways”, ‘rural training pathways’, ‘rural training programme’, ‘rural education’ ‘rural exposure’, ‘rural practice’, ‘rural experience’, ‘rural attraction programme’, ‘rural scholarship programme’; “return to service” programme, rural placement programme, rural retention programme, rural employment programme, rural development programme, or bundled interventions.
- Context: SSA countries according to WHO countries list.

We also used literature search through WONCA (World Health Organization of Family Doctors) websites and Google. Additionally, we used iterative snowball or citation tracking techniques—that is reviewing reference lists of primary included articles to identify additional relevant studies. These articles were assessed between November 2021 and February 2022.

The principal investigator (DK) carried out the literature search. RvdP reviewed search strategies. All selected references were either saved using PubMed functions, or on Mendeley desktop software.

### Study selection

Our search results were cleaned for duplicates by two members of our research team (DK and RvdP) using following eligibility criteria: (i) studies describing rural pipeline strategy or pillar; (ii) studies describing facilitators or challenges of rural pipeline implementation; (iii) studies describing the impacts of the rural pipeline intervention on local health services and communities (e.g. quality of care, health coverage, etc.). Discrepancies in study selection processes were resolved by a discussion between the two reviewers.

### Data charting

Data, mainly qualitative, were extracted by the two reviewers (DK and RvdP) using an Excel spreadsheet. This Excel form was piloted on three articles before use. Data collected included: (i) study characteristics—authors name, year of publication, study country and study design; and (ii) information related to implemented interventions—implementation strategies, facilitators or barriers of rural pipeline programmes implementation, and programmes outcomes or effects. Given our research questions, both quantitative and qualitative data analyses were done.

### Summarizing and reporting results

Characteristics of included studies were summarized using descriptive statistics. We then used thematic content analysis to sum up the main findings of the review. We reported the results following the PRISMA Extension for Scoping reviews guidelines [35].

## Results

### Description of studies included in the review

**Fig. 1:**
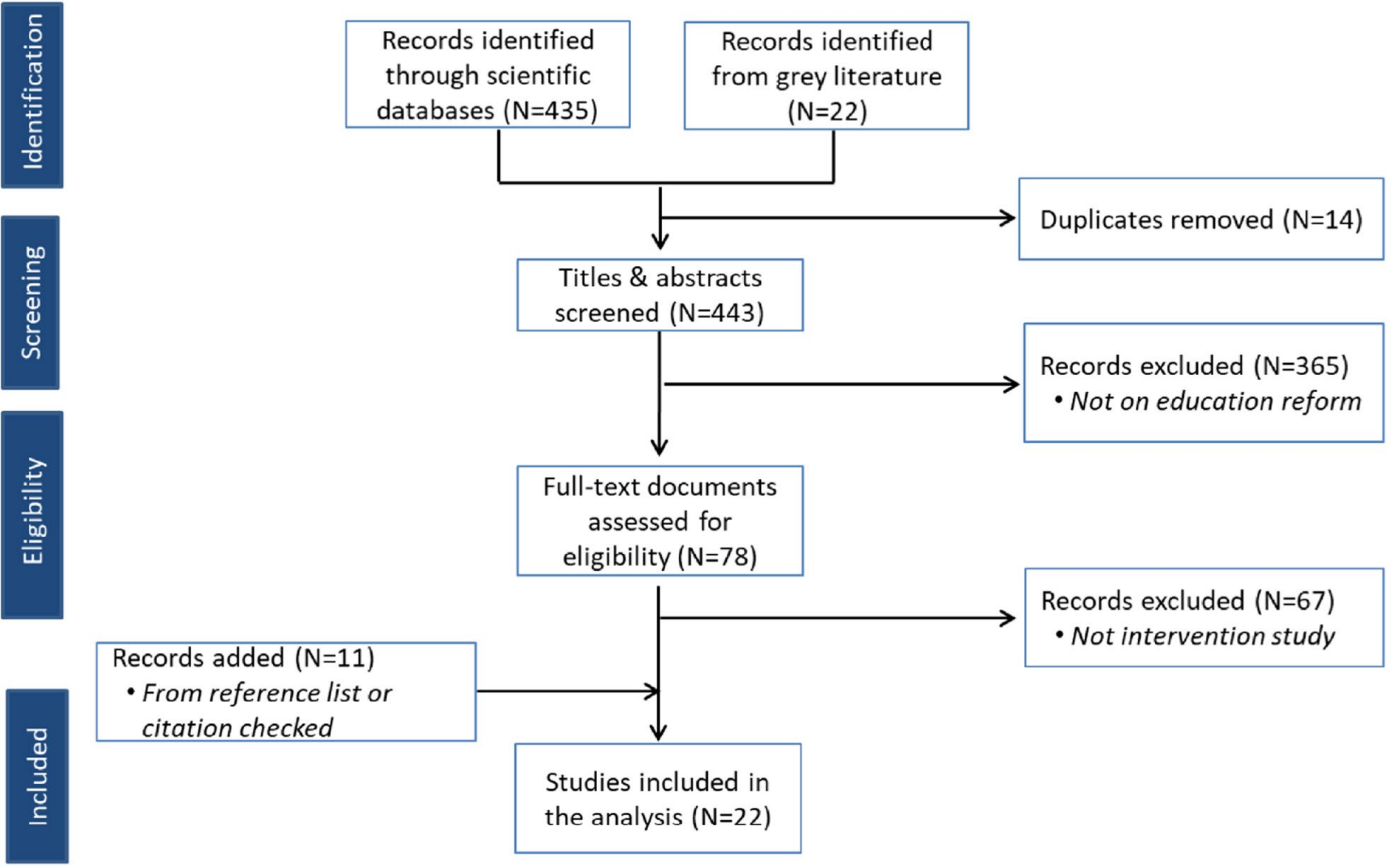
Study selection process

The present review includes 22 studies published between 2007 and 2021, with the majority (fifteen) published from 2015 [29–31, 36–47]. Figure 1 shows the studies selection process. These studies were from eight countries, with South Africa (eight studies [30, 39–41, 44, 47–49]), Uganda (four studies [23, 24, 43, 46]), Ghana (three studies [36–38]) and Mali (three studies [25, 26, 31]) being the most represented. One study was reported from each of the following countries: Malawi [28], Tanzania [42], Bostwana [45] and the Demographic Republic of Congo [29], DRC (Table 1). These were predominantly qualitative (nine studies [23, 24, 26, 29, 30, 44, 46, 47, 49]) and quantitative (five studies [37, 38, 40, 42, 43]) methods studies. Eight studies used a mixed-methods approach [25, 28, 31, 36, 39, 41, 45, 48].

**Table 1:** Characteristics of studies included

Authors, title, year, country	Study participants	Study design	Pillars of the rural pipeline programme					Rural pipeline programme implementation		
			Advocating health professions among rural students	Ensuring that more rural students are selected into programmes	Developing a curriculum oriented towards rural health training and delivery	Ensuring rural retention through educational and professional support	Other pillar	Facilitators	Challenges	Outcomes
Sidibé et al., Rural pipeline and willingness to work in rural areas: mixed methods study on students in midwifery and obstetric nursing in Mali, 2019, Mali	Students in midwifery and obstetric nursing	Mixed-methods	Decentralization of medical schools to rural and underserved locations Entrance test dates are announced and published on radios	Organization of tests in all regions, including rural and underserved regions Allocation of scholarship covering tuition and living expenditures	Existence of a mandatory policy exposing students to rural practices Clinical practices in rural communities during internship	[Not reported]	[Not reported]	No age restriction for students applying to training programme Availability of schools in rural settings	Most candidates from urban areas Admission to private schools is conditional to tuition fees payment Lack of support for subsistence of private school students during internship in rural area Inadequate equipment, poor road network and housing conditions	Current living location in rural areas, having professional school, and having had secondary education in rural areas are greater intention to go to rural areas
Amalba et al. The effect of Community Based Education and Service (COBES) on medical graduates’ choice of specialty and willingness to work in rural communities in Ghana, 2016, Ghana	Medical students	Cross-sectional	[Not reported]	[Not reported]	As part of the Community-based Education services (COBES) programme, for the 3 first years, students are sent to communities, with at least a primary health care facility Each of these 3 years, students spend four weeks in the community in groups of 8–10 students per community Students are expected to identify and explain factors (e.g. demographic, economic, social, political and environmental) that affect the community health, and subsequently prioritize them and identify the resources available in the community to contribute to meeting those needs Students take turns to rotate through the various sections of the health facility (e.g. for example, dispensary, consulting rooms, MCH clinic, laboratory, and immunization programmes)	[Not reported]	[Not reported]	[Not reported]	Scarcity of medical schools in rural areas	Programme develops graduates to adapt to rural lifestyle; which results in high future intention practice in rural areas Graduates learn the culture and lifestyle of the community when they interact with community members; hence their willingness to go back to the community after graduation becomes strong
Hatcher et al. Placement, support, and retention of health professionals: national, cross-sectional findings from medical and dental community service officers in South Africa, 2021, South Africa	Medical doctors and dentists	Cross-sectional	[Not reported]	[Not reported]	A year of compulsory community service for young professionals The programme started with medical doctors, dentists and pharmacists and grew to include physiotherapists, clinical psychologists, dieticians, radiographers, environmental health practitioners and nurses	[Not reported]	[Not reported]	Graduates reported greater satisfaction with supervision, management, and practical concerns during the community service year. In terms of supervision and management, most felt well-oriented to the job (87%) and reported that seniors were available when needed (82%). Approximately three quarters felt they had good clinical supervision (71%) as well as satisfactory mentorship and support (72%)	Approximately half reported accommodation as unsatisfactory (43%), their personal safety as lacking (66%), and remuneration as unfair (46%)	More than half (55%) of graduate were placed in rural areas High proportion of health workers intended to remain at the same facility (34%) and one quarter (25%) intended to work in rural and underserved communities Among those who intended to leave, 68% intended to specialize
Amalba et al. Training preferences regarding choice of place of work after completing medical training in traditional or problem-based learning/community-based education and service curricula: a study in Ghanian medical schools, 2019, Ghana	Medical students	Cross-sectional	[Not reported]	[Not reported]	Similar to what is described above (COBES programme)	[Not reported]	[Not reported]	[Not reported]	Country medical education mainly concentrated in urban cities Inadequate infrastructure of health facilities in the rural communities Rural outreach programmes not incorporated in curriculum Discriminatory admissions policy for students in rural areas	Increased willingness to practice in rural area Male students were 1.5 times more likely to indicate that they were willing to work in rural areas after graduation compared to their female counterparts Students who had lived in rural areas prior to entering medical school were almost four times more likely to choose to work in a rural area after graduation than those from the city
Clithero-Eridon et al. Future medical student practice intentions: the South Africa experience, 2020, South Africa	Medical students	Cross-sectional	[Not reported]	[Not reported]	[Not reported]	[Not reported]	Social accountability principles guide the rollout of this strategy The Network Towards Unity for Health (THEnet) is used to measure progress towards meeting the educational component of this vision including examining socio-demographic and practice intention characteristics. For example, with the THEnet methods, students selected into the training programme should demographically mirror the population they are expected to serve	[Not reported]	[Not reported]	One-third (34%) of respondents indicated an intention to go into primary care, A similar proportion of female (36%) and male respondents (38%) indicated a plan to enter primary care Less than one-third of students (27%) indicated they would go abroad after graduation Reasons for leaving was to gain experience and the belief that there were better opportunities overseas Black African respondents were significantly less likely to state an intention to work abroad than other respondents
Gumede et al. Engaging future healthcare professionals for rural health services in South Africa: students, graduates and managers perceptions, 2021, South Africa	Medical students	Qualitative study	[Not reported]	Intervention invoking an integrated model of students’ recruitment at school level through selection by local health facilities Allocation of scholarships to disadvantaged students in rural provinces	[Not reported]	Upon completion of their degrees, graduates are absorbed into the hospitals where they were initially interviewed for the scholarship The selected graduates sign a year-for-year work-back contract with programme	Social mentoring programme that aims to prepare students for life at university and away from home, and to cope during difficult times	[Not reported]	[Not reported]	Programme brakes the cycle of poverty for needy students, by introducing career guidance, informing them of work opportunities in the healthcare field, and making it possible for them to complete the necessary courses Students had become role models for rural youth; local graduates act as a point of reference for youth who do not value education and drop out of schools Patients can now access health services without needing interpreters to convey the message to the health care practitioners Improved communication with grassroots communities as students/graduates speak local language; they understand the community needs and are willing to go the extra mile to provide quality healthcare. This not only helps to produce a stable workforce but makes it possible for the hospitals to have outreach services to prevent the spread of diseases in the community
McGregor et al. The socio-economic impact of rural-origin graduates working as healthcare professionals in South Africa 2019, South Africa	Medical doctors, nurses, dentists, pharmacist, etc,	Mixed-methods	[Not reported]	Selection of rural origin students from poor family Allocation of scholarship to students from rural areas	Graduates are expected to return to the district hospital where they were selected to fulfil a year-for-year work-back obligation	[Not reported]	[Not reported]	Available of funding Support of national Stakeholders including local communities	Poor infrastructure, inadequate human resources and reduced teaching capacity of schools located in rural areas	From 1999 to 2016, 337 rural-origin students were trained In 2017, 145 health professionals (out of the total 337 graduates) had completed their work-back obligation; 63% of them were still working in rural health facilities Health professionals trained under this programme witnessed major changes in their household economic and social circumstances as soon as they started working as health care professionals It costed 184 million South Africa Rand ZAR Rand, ZAR (equivalent of 11.5 million USD) to train 254 graduates. These graduates’ lifetime earnings were estimated at 4 billion ZAR (251 million USD)
Kapanda et al. Enhancing future acceptance of rural placement in Tanzania through peripheral hospital rotations for medical students, 2016, Tanzania	Medical students	Cross-sectional	[Not reported]	[Not reported]	Exposure of medical students to rural working environments; in 2012 a 12- week peripheral hospital rotations initiative was introduced for third-year medical students	[Not reported]	[Not reported]	[Not reported]	Most secondary schools in rural areas are ill-equipped with laboratory spaces and lack science teachers to prepare them for admission in medical schools; this disadvantage rural students in selection processes by medical schools Poor infrastructure in rural areas (lack of electricity, poor roads, and general poverty of rural populations) hampers the establishment of medical schools in rural settings, and as a consequence most medical schools are located in urban centres	Students exhibited positive attitudes toward peripheral hospital placements Students with male gender, aged 25 years or above, enrolled after health-related practice (in-service), born in rural area, being satisfied with peripheral hospital placement were likely to accept deployment in rural locations after graduation
Kizito et al. Influence of community‑based education on undergraduate health professions students’ decision to work in underserved areas in Uganda, 2017, Uganda	Medical students	Before-and-after study	[Not reported]	[Not reported]	As part of the Community-based Education, Research and services (COBERS) programme, students are sensitized and acclimatizes to working in underserved communities and to enable them acquire the appropriate attitudes towards working in these areas COBERS is compulsory for medical, nursing, dentistry, pharmacy, biomedical engineering, cytotechnology, radiography and medical laboratory sciences students	[Not reported]	[Not reported]	COBERS was established with funding from the US Government through the Medical Education Partnership Initiative and technical support from Johns Hopkins University	[Not reported]	After the COBERS placement, factors that were significantly associated with choice to work in the rural areas included access to long distance, and being posted in the rural areas by the ministry Factors associated with intended duration of work in the rural areas after students had undergone a COBERS placement included university attended, availability of reliable electricity, access to long distance medical courses, and having the intention to work in another African country
Couper et al. Influences on the choice of health professionals to practise in rural areas, 2007, South Africa	Medical doctors	Mixed-methods	[Not reported]	Priority selection of students from rural origin	Exposure of students to rural health delivery during training and education	[Not reported]	[Not reported]	[Not reported]	[Not reported]	Decision to work in rural area is underpinned by personal attributes, including: serving people (especially one’s ‘own people’), having a community connection, wanting to serve people in rural areas and the need to make a difference and have an impact For those of rural origin, the greatest motivating factors was sense of returning home, and of familiarity with and ability to relate to rural people, coming back to roots, family, people, and village and being born there Some felt a sense of obligation, needing to give something back to the community which had nurtured and supported them, because ‘my success is the community’s success’
Mapukata et al. Factors influencing choice of site for rural clinical placements by final year medical students in a South African university 2017, South Africa	Medical students	Explanatory qualitative study	[Not reported]	[Not reported]	A Wits (Faculty of Health Sciences at the University of the Witwatersrand) programme was launched in 2003 as a 4-year training programme that complemented the existing traditional approach to medical training, with both streams being combined in the clinical years. Within that, the integrated primary care block, which is a compulsory 6-week placement in a range of primary health care settings, was launched in 2006 as one of the initiatives that would strengthen the university’s and students’ commitment to rural and underserved communities. Through the IPC block, district facilities in underserved communities of four provinces (Gauteng, North West and Mpumalanga) provide the context for final year medical students to achieve some of the core competencies of an integrated curriculum	[Not reported]	[Not reported]	Good supervision was reported to influence students’ choice of rural sites Existence of comfortable accommodation, and provision of three meals per day at no cost to the student	[Not reported]	[Not reported]
Mpofu et al. Impact of an interprofessional education programme on developing skilled graduates well-equipped to practise in rural and underserved areas, 2014, South Africa	Medical students	Mixed-method	[Not reported]	[Not reported]	The Faculty of Community and Health Sciences (FCHS) at the University of the Western Cape (UWC) has, therefore, developed a specialized South African Inter-Professional Education (IPE) programme that provides health sciences students with structured learning opportunities that combine service learning with teamwork and reflection The programme offered required students in the disciplines of Natural Medicine, Physiotherapy and Nursing in their third and fourth years to address collaboratively identified community priorities. These priorities were identified by UWC together with community and health service providers Students were expected to work in interdisciplinary teams and initiate community interventions for the identified priorities. They were engaged in a process of structured reflection during the IPE course and delivered a presentation to the community at the end of the placement. Students were supervised once a week according to their disciplines by selected academics with the requisite knowledge, skills and experience, as well as by on-site professionals working in the practice settings	[Not reported]	[Not reported]	[Not reported]	[Not reported]	The majority of the students (69%) preferred to work in rural-based communities with less than a third of the group preferring to work in urban-based communities Students indicated that the need for health promotion in the community and the community’s friendliness motivated them to want to practise in the rural areas and to work there in future Some students want to work in rural areas in order to address the problem of limited resources Other students also felt that their personal needs, such as their desire to earn more money, would not be satisfied in a rural setting
Tonya et al. Rural exposure during medical education and student preference for future practice location—a case of Botswana, 2016, Botswana	Medical doctors	Mixed-methods	[Not reported]	[Not reported]	Exposure of students to rural health care delivery throughout a 5-year curriculum Rural rotations of students during their training curriculum	[Not reported]	[Not reported]	[Not reported]	94% of graduates perceived poor opportunities for career advancement Career stagnation, lack of learning opportunities, and professional isolation Absence of monetary compensation in a context of rural isolation, lack of basic infrastructures (eg: regular water supply, good schools for children)	The majority (91%) of graduates wanted to practice in urban areas. However, 31% said it was likely/very likely that they would practice in a rural area Rural rotations showed them that practising in a rural area is not as difficult as previously perceived Students with a rural background were more likely to practice in a rural area if it was near their home towns
Strasser et al. Increasing nursing student interest in rural healthcare: lessons from a rural rotation programme in Democratic Republic of the Congo, 2021, RD Congo	Nurses	Case study	During the alignment phase of the rural rotation (RR) programme, meetings were conducted with the Ministries of Health and Education, Nursing Council, Midwife Association, target schools, students, and students’ parents to raise awareness of the subject by explaining to them the requirements of Education Reform using a competency-based approach: train students to be future health workers on a global scale; and prepare them to work in all conditions, even in the remote environments both in hospitals and in rural communities	[Not reported]	The RR programme aimed to increase nursing students and community health worker (CHW) exposure to rural health needs as well as rural clinical practice and community engagement prior to graduation, entry to practice, and employment The programme also focused on nurses’ readiness for clinical practice through curricula reform and development of innovative pedagogy including use of skills labs and simulation-based training	The RR programme included the development of a tele mentoring programme to bridge rural health centres with urban specialists and trainers as well as assistance with national registration of nurses	[Not reported]	Availability of PEPFAR funding to support HRH strengthening in DRC	Lack of financial support for students, poor housing and living conditions in rural areas	93% of students agreed or strongly agreed that they would recommend RR participation 97% agreed or strongly agreed that their RR had strengthened their educational experience 95% of students agreed or strongly agreed that they felt better equipped to provide HIV/AIDS prevention, care, and treatment services after RR participation When asked about their biggest RR challenges, students most reported financial support (35%), housing (30%), and rural living conditions (3%)
Kelly et al. Predictors of Workforce Retention Among Malawian Nurse Graduates of a Scholarship Programme: A Mixed-Methods Study, 2015, Malawi	Nurses	Mixed-methods	[Not reported]	Global AIDS Interfaith Alliance offers preservice scholarships for nurses who need assistance with college fees and who demonstrate a commitment to work in the public health system after graduation The scholarship programme supports nursing students who are predominantly orphans or from lower socio-economic backgrounds; the scholarship recruitment phase targets needy students who have been accepted to nursing college (including one located in and serving a rural area) who are facing financial hardship and would otherwise be unable to complete a diploma/degree in nursing	[Not reported]	GAIA maintains close follow-up with each scholar through site visits, text messaging, phone calls, and regional get-together events, providing academic assistance, psychosocial and clinical mentoring, and educational support, including opportunities for continuing professional development. GAIA staff maintain follow-up data on all graduates in a continually updated database GAIA’s follow-up activities assist the Ministry of Health with tracking whether GAIA scholars have reported to deployment sites	The scholarship programme aims to increase the number of nurses working in the public sector in 2 ways: by providing tuition support, living stipends, uniforms, nursing supplies, and payment of council exam fees to improve yearly school progression of nursing students, on-time graduation rates, and licensing exam performance; and by requiring students to sign a service agreement to work for the Ministry of Health for 4–5 years after graduation from nursing school to retain them in the public sector	Feeling appreciated by their supervisors and emphasized positive relationships with co-workers as factors associated with remaining in their current job Job security, continuing education, and public service agreement	Lack of resources and high nurse-to-patient ratios Poor working relationships with management or other nurses Low salaries in relation to workload, poor housing options, and lack of appreciation	Most of the graduates were working in urban areas (67% urban, 23% rural, and 10% peri-urban)
Amalba et al. The perceived usefulness of community-based education and service (COBES) regarding students’ rural workplace choices, 2016, Ghana	Medical students	Mixed-methods	COBES aims to create awareness among students of the importance of developing community partnerships as a means to implement sustainable healthcare initiatives	[Not reported]	On yearly basis students spend four weeks in the community with predefined objectives until year four. In year five and six, they are scheduled for community posting at district hospitals	[Not reported]	[Not reported]	Existence of partnerships between the university, service providers and community as well as the students’ learning and service activities, positively influences and prepares students to care for people in the rural communities	Insufficient motivation of staff and lecturers to spend adequate number of days in guiding the students in the communities The lack of basic equipment at the facility level	The community sees the relevance of health promotion and education. As the students give talks on health education and carry out health promotion activities, the behaviour of the community, as well as their health seeking behaviour changes and their awareness towards health and their knowledge on health issues improve The presence of the students in the community serves as motivation for the youth. In the northern part of the country where most families do not see the relevance of children’s education, parents do not invest in their education. Encountering female students may convince parents that educating the female offspring can be very rewarding Participants indicated that the presence of students in the community provides workforce to the community. They are also able to identify the needs of the community and propose solutions with the support of the community members Participants alluded to the fact that students do benefit a lot from COBES activities in the communities. Stakeholders acknowledged that the community serves as a learning platform where students interact with people of different cultural backgrounds. This helps them improve their communication skills, help to build their clinical and social skills and empowers them in their clinical work COBES helps students get a clear understanding of primary health care setting within the health structure. Having part of their training in the community helps them to make choices as to which areas they want to specialize and also develop interest to practice in the rural area after graduation Some of the health facility staff were of the opinion that since most of those who guide the students in the community are community health nurses, the University, as a way of incentive, could offer some of them admission into the University to pursue further studies to better guide the students who are more knowledgeable than them
Atuyambe et al. Undergraduate students’ contributions to health service delivery through community-based education: A qualitative study by the MESAU Consortium in Uganda, 2016, Uganda	Medical students	Qualitative study	[Not reported]	[Not reported]	The programme exposes students to health delivery in rural communities Before students go to the sites, they are briefed and are given overview lectures that introduce them to community health, Primary Healthcare and what to expect during their COBERS attachment	[Not reported]	[Not reported]	Uganda had, in 2016, 6 preservice medical training institutions. Five of them (Universities of Gulu, Makerere, Mbarara, Kampala and Busitema) came together to form Medical Education for Equitable Services to All Ugandans consortium (MESAU) with funding from the US Government supported Medical Education Partnership Initiative (MEPI) and technical support from Johns Hopkins University	Inadequate, and in some cases total absence of transport for outreaches to communities was a key constraint to students’ activities and to reach at some sites In some cases, medicines and other supplies like gloves were limited which meant that students could not effectively meet the demand in the communities	Students were not only learning; they were also contributing to health service delivery Students’ contribution at the health facilities was described in various ways which we grouped into five categories during analysis. Students were described as: being caring and compassionate, available on time and anytime, participating in patient care, willing to help and share their knowledge and skills, and stimulating discussion on various topics in health as well as inspiring health workers regarding work ethics COBERS students participated in various community health activities in the areas of water and sanitation and hygiene. Students contributed to maintenance of safe water sources, educated communities on drinking safe water in the households and on good sanitation practices including latrine construction, hand washing and appropriate waste disposal. Hygiene was promoted at household level and at community level for example among food handlers in markets. Public health education extended to institutions such as schools where sensitization on various health-related issues including sexuality and sexual health was conducted Students presented extra workload for some health workers (supervisors) Other health workers reported that they spent more time on each patient because they had to explain to students as they provided patient care Some patients did not appreciate being attended to by students with the effect that the number of mothers coming to the facility for delivery during COBERS placement was reduced Some health workers (supervisors) perceived the presence of COBERS students as an opportunity to take unofficial leave thus leaving students unsupervised
Kaye et al. Influence of the training experience of Makerere University medical and nursing graduates on willingness and competence to work in rural health facilities, 2010, Uganda	Medical students, nurses	Participatory approach	[Not reported]	Allocation of social amenities and affordable cost of living to students from rural areas	[Not reported]	[Not reported]	[Not reported]	Availability of social amenities and affordable cost of living; ease of communication (no language or cultural barriers); personal safety and security considerations; the opportunity for career advancement; and considerations about workload	Inequitable and poor remuneration, overwork due to understaffing, having no time for holidays, and the overwhelming responsibilities of clinical care, planning and administration in the context of limited resources or prior experience work environment lacking stimulation and characterized by inadequate supplies, equipment and support supervision from the ministry of health or district officials, and low access to continued professional education	Community based training was identified as the main factor shaping the values and attitudes of those who were in favour of rural practice, and were confident and willing to work in a rural area. The interaction of medical and nursing graduates with the community during the community based training curriculum appeared to prepare trainees for rural practice by changing their attitude to working in a rural area
Kaye et al. Perceptions of newly admitted undergraduate medical students on experiential training on community placements and working in rural areas of Uganda, 2010, Uganda	Medical students, nurses	Qualitative study	[Not reported]	[Not reported]	Experiential training on community placements and working in rural areas	[Not reported]	[Not reported]	[Not reported]	Whereas only about one third had their home district in the central region, over 3 out of 4 completed their high school from schools located in the central region, and possibly were not conversant with rural areas Being cut off from friends and colleagues, absence of guidance from faculty or any tutors and inadequate exposure to the variety of conditions, which exist in the large teaching hospital Inadequate support facilities like internet and libraries to enable self-directed learning, and inability to understand the local languages or cultures	This community-based education programme was reported to enable students understand the medical conditions in rural areas, to see a variety of medical conditions (some of which are not seen in the teaching hospital), and to learn about the management of the health care system
Schalkwyk et al. Consequences, conditions and caveats: a qualitative exploration of the influence of undergraduate health professions students at distributed clinical training sites, 2018, South Africa	Medical students	Qualitative study	[Not reported]	[Not reported]	In recognition of the need for more socially relevant training, undergraduate health professions students were sent to district and community health facilities (within the public healthcare system) as part of their clinical exposure	[Not reported]	[Not reported]	[Not reported]	[Not reported]	Having students at the site manifested in the organizational culture and the ways in which staff at the facilities engaged with one another. For example, respondents spoke about the way in which bringing the academic endeavour to the facility had a ripple effect on the culture in the facility by encouraging the adoption of a more evidence-based approach Students were involved in. In the health facility, for example, they assisted with history taking and examination of patients, the clerking of patients, dealing with emergencies, and performing certain simple procedures. The allied health students provided therapeutic interventions. In the community context, students conducted. Quality Improvement and Community Oriented Primary Care projects, and were involved in health promotion, patient follow-up and home visits. In performing these clinical activities, students added to service delivery by doing more in-depth as well as comprehensive assessments of patients, being able to see patients more regularly than qualified clinicians, and assisting facility staff in patient care Supervisors enjoyed the opportunity to interact with students. This was often expressed in terms of conveying their own enthusiasm and interests to future health care professionals
Dormael et al. Appropriate training and retention of community doctors in rural areas: a case study from Mali, 2008, Mali	Medical doctors	Participatory action research	[Not reported]	[Not reported]	[Not reported]	In response, the NGO and the Rural Doctors Association decided to set up an orientation course for recently established rural doctors. The underlying assumption was that training meeting rural practitioners' needs would strengthen young doctors' technical competences and self-confidence, and consequently contribute to retention Rural practice is promoted through a package of non-financial incentives provided by an NGO (Santé Sud) and the Malian Rural Doctors Association: young doctors settling in rural areas usually benefit from interventions aiming at improving living conditions (water, solar panels, motorbike) and working conditions (basic equipment, continuous education, peer support and mentoring)	[Not reported]	[Not reported]	They reported frequent relational problems detrimental to their social integration: conflicts with the health centre committee (their employer) about working conditions and financial management issues, leadership conflicts with other staff members, absenteeism and misbehaviour of staff, tense coexistence with traditional practitioners, or disagreements with the district medical officer concerning boundaries between first line care and hospital care	Between 2003 and 2007, 65 newly installed rural doctors, deployed in all regions of the country, participated in the training. Table 3 shows yearly cohorts and retention in rural practice over the years. At the end of 2007, 55 out of the 65 trained young doctors (85%) were still engaged in rural practice Regarding the three first cohorts trained in the period 2003 to 2005, respectively, 50%, 77% and 86% were still in rural practice 4, 3 and 2 years after the training. Eight out of 32 trainees for this period were no longer in rural practice end of 2007; five of them left within the two first years of installation. The 8 "dropouts" went for specialist training, got involved in a private practice in the capital city Bamako, or were hired by an NGO
Coulibaly et al. Une médecine rurale de proximité: l'expérience des médecins de campagne au Mali, 2007, Mali	Medical doctors	Mixed-methods	[Not reported]	[Not reported]	[Not reported]	Prior training is offered to young people setting up, as well as regular follow-up. The support of the professional association plays a crucial role in the fight against professional isolation: regional and national meetings, continuous training, mutual visits, etc. Several factors, both financial and non-financial, contribute to the acceptability of working in rural areas. Young doctors are encouraged by professors from the Faculty of Medicine. The Santé-Sud project supports them by providing an installation kit, solar panels and a motorbike	[Not reported]	[Not reported]	[Not reported]	The presence of a doctor increases the use of services. The utilization and coverage rates of preventive activities of centres run by eight experienced rural doctors were higher than those of other centres in the same districts. Some centres with doctors attract many patients from outside their area A reduction of more than 80% in the number of epileptic seizures of patients followed by the field doctors. Prior training is greatly appreciated by young doctors, who say they are better prepared to face field practice A relative stability of rural doctors: 45% had been practising for more than 5 years, and 25% planned to practise for more than 10 years

### Pillars and impacts of rural pipeline programmes

One more rural pipeline pillar (social accountability) was identified in addition to the four reported by Tesson et al. [10] (advocating health professions among rural students; ensuring that more rural students are selected into programs; developing a curriculum oriented towards rural health and rural exposure during training; curriculum oriented to rural health delivery, and ensuring retention of HWs in rural areas through educational and professional support). We followed the above six pillars of the rural pipeline to report the results; this is why the focus of the paper in the sections that followed may varies from lines to lines, depending on which pillar is being described.

#### Advocating health professions among rural students

This pillar of the rural pipeline programme includes three main strategies reported from different countries. The first strategy, reported from Mali, is the decentralization of medical schools to the regions (cities in the countryside including rural locations) to favour local medical training [31]; the second, also from Mali, is using radios to announce dates for the start of school programmes [31]; the third strategy was reported from DRC, Ghana and Uganda, and is related to raising awareness of students and their parents about the need and importance of education reform that promote rural pipeline [29, 36, 37, 43].

None of the selected studies for this review directly reported impact of this pillar on health, and socio-economic situations.

#### Ensuring that more rural students are selected into programs

For this pillar, two strategies have been reported. In Mali, this consisted of organizing admission tests for medical studies in all regions [31]. In Malawi and South Africa, the strategy was to grant scholarship to needy students; that is mostly those with a low socio-economic status [28, 30, 41]. For instance, in South Africa, the scholarship programme increased the number of low socio-economic students enrolled from four (4) in 1999 to 337 over 2017 [30, 41].

This pillar has been documented to favour three impacts. First, it helps to increase the number of rural health practitioners; in Mali, enrolling in medical training programmes students who had secondary school in rural areas was reported to favour health practitioners’ intention and willingness to work in rural areas [31, 41]. Second, this pillar contributes to reducing communication barriers between healthcare providers and community members. Gumede et al. in South Africa have mentioned with the employment of local health practitioners, rural patients access health services without needing interpreters to convey the message to the healthcare practitioners [30]. Practitioners better understand the community needs and provide quality health care. In addition, the lift of communication barrier favours outreach services in course of which practitioners communicate to prevent the spread of diseases in the community [30].

Third, this pillar is reported to contribute to socio-economic development of rural areas. McGregor et al. and Gumede et al. in South Africa reported major changes in household economic and social circumstances of students from poor family after their graduation [30, 41]. Compared to their parents and families were they grew up, graduates were able to buy cars, build decent family houses, send their children to private schools and take care of their siblings [41]. The return on investment of this program in rural South Africa was estimated to be 22 times the cost of the training [41].

#### Developing a curriculum oriented towards rural health training and delivery

This pillar consists of two main strategies: emphasizing primary care throughout the curriculum [40] and placing students in rural communities and health care settings. Regarding the curriculum, Amalba et al. in Ghana and Strasser et al. in DRC documented that students, before graduation, were trained on how to do community health diagnosis and identify available resources to meet the needs, but also to understand functioning of rural health facilities [29, 37]. In Uganda, Atuyambe et al. said that just before sending students to communities, they are briefed on community health and primary health care at school [46].

For the placement of students in rural communities and health care settings, Amalba et al. reported that the Ghanaian context was marked by Community Based-Education services (COBES) that consisted of dispatching groups of 8–10 students to rural communities and health care settings for four weeks every year, during three consecutive years [37]. In Tanzania and South Africa, students were sent to communities and primary healthcare settings for twelve weeks and six weeks, respectively, each year [42, 44, 47]. In Mali and Bostwana, this pillar consisted of adopting a mandatory last year rural training policy for students. In public and private schools, students at the final year of graduation were required to do practical training in a rural environment, as part of the examination process [31, 45]. The training was planned for 45 and 56 days, in Mali and Bostwana, respectively, under the direct supervision of rural health care providers [31, 45]. In Mali, this training was concluded with a report produced by the student and approved by the teachers [31]. In South Africa, Hatcher et al. reported that a year of compulsory community service was adopted for young health professionals, including medical doctors, dentists and nurses [39]. In South Africa, a similar approach of compulsory service was reported by McGregor et al. whereby graduates who benefit a scholarship during their training were expected to fulfil a year-for-year work-back obligation in rural areas [41].

Six impacts—of which four at the health facility level and two at the community level—are reportedly related to this pillar.

At the facility level, this pillar contributes to increasing rural health practitioners. In Ghana, Amalba et al. found that making students follow a curriculum integrating exposure to rural training and practice increased their willingness to work in rural areas [38]. This pillar also reinforces professional development of health practitioners; on the one hand, the communities to which the students are sent serve as learning platforms where they interact with different cultural backgrounds. Through this way, they improve their communication skills, build their clinical and social skills and empower themselves in their clinical work [36]. On the other hand, sending students to rural care settings for practical training commit senior rural health practitioners to guide the students. This may lead the University to incentivize the senior practitioners by offering some of them admission into the University to pursue studies to better guide students who are acknowledgeable than them [36]. The third impact prospect attributed to this pillar is the improvement of the quality of health services; in South Africa, young practitioners or students deployed in the care settings were told to improve the service delivery by doing more in-depth as well as comprehensive assessments of patients, being able to see patients more regularly than qualifies clinicians [30, 47].

However, increase in rural health workers’ workload and supervisors’ absenteeism rate have been reported as unexpected impacts of this pillar. First, Atuyambe et al. in Uganda reported the placement of students in rural facilities as an extra workload for the rural health workers, as the latter are assigned to supervise students’ practices [46]. Indeed, with the presence of students, senior health workers have to explain to them the practice as they provide care to patients. Besides this being more time consuming, it results in fewer patients being delivered care per day. Second, Atuyambe et al. in Uganda found that some HWs use the presence of students in health facilities as an opportunity to take unofficial leave, hence leaving students unsupervised [46]. This practice was reported to hamper students’ learning capacity.

At the community level, the deployment of medical students into rural communities for practical training has been reported in Ghana and Uganda to contribute to health education and promotion within these communities. Students’ activities in rural communities included communications on drinking water, hand hygiene, good sanitation practices including latrine construction, sexual health and other health issues [36, 46]. Students in Uganda were also told to contribute to the maintenance of safe water sources [46]. In addition, the presence of medical students in rural communities has been documented to motivate the youth who see these students as role model. Indeed, with the presence and perceived usefulness of these medical students, community members realize the relevance of children’s education, thereby encourage them to enrol their children in school [37].

Nonetheless, patients’ discomfort of being attended by students has been reported as an impact of this pillar. A study in Uganda reported that some health managers had difficulties convincing community members to be attended by students as they perceived students as not qualified to provide care [46]. This attitude of communities towards students has been reported to influence health facilities attendance, especially for maternal health services such as birth delivery [46].

#### Ensuring retention of health workers in rural areas through educational and professional support

This pillar includes four aspects to account for. The first aspect is to ensure that rural health facilities are well equipped to allow smooth functioning of the facility, provision of quality care, thereby allow job-satisfaction of the staffs [30, 31]. This training would strengthen young professionals’ technical competences and self-confidence [25, 26]. The second aspect is to ensure continuous training to meet rural practitioners’ needs. Thirdly, retaining HWs in rural areas requires conducting regular field supervisions to assess, acknowledge, support and encourage rural health practitioners’ work [25, 28]. The fourth aspect is the organization of regular national/regional encounters or reciprocal visits between rural and urban health practitioners to fight professional isolation [25, 28]. Such encounters can be promoted by health professional associations [25].

This pillar has been reported to increase the number of rural health workers; training health practitioners in primary health care was said to retain them in this location. Dormael et al. in Mali reported that 55 out of 65 trained young doctors (85%) deployed in rural areas remained at their location, two years after the training [26]. In addition, retaining health workers in rural areas was reported to yield in social development of health practitioners as they learn the culture and lifestyle of the community, and benefit from the hospitality of community members [38]. Furthermore, some practitioners got married or engaged to local people, thus fulfilling a social endeavour [30].

#### Ensuring social accountability

Clithero et al. in South Africa reported about social accountability of health professionals as an important part of the rural pipeline [40]. This consists of ensuring that the students enrolled in the training programmes will meet communities’ health needs after graduation. In the South African context, as specific indicators to assess this, education authorities checked whether the students being enrolled have the socio-demographic profiles matching the populations to be served and whether these students choose to practice primary care and intend to practice in areas of high need such as underserved and rural areas. Another strategy for social accountability has been described by Gumede et al. in South Africa [30]. It is to apply an integrated model of student recruitment, which involves enrolling student by local health facility, grating them a scholarship, exposing them to a compulsory structured academic and social mentoring programme, making them attend experiential holiday work at the health facility, and absorbing them into the health facility through which they were enrolled. A complementary approach for this pillar, reported by Kelly et al. in Malawi, is to ensure that scholar students fulfil the service agreement after graduation [28]. In the case of Malawi, the funding agency maintained a close follow up with each scholar through site visits, text messaging, phone calls [28].

None of the selected studies for this review directly reported impact for this pillar.

### Factors influencing rural pipeline implementation

Ten studies reported about drivers in implementing rural pipeline program in sub-Saharan Africa [23–25, 28, 30, 36, 37, 39, 41]. Seven main drivers emerged from these studies, including availability of medical school and health facility infrastructures in rural areas, assessment of readiness of rural clinics for students’ practical training, mapping of rural sites for students’ accommodation, setting no age restriction for students applying for rural medical schools, appropriate academic capacity-building programmes for medical students, motivational dynamics for students to do their career in rural settings, students’ personal assets for and interest in rural practice, and locking health personal recruitment in urban area.

Availability of medical school and health facility infrastructures has been mentioned in studies from Mali, Uganda and DRC as important facilitator in implementing rural pipeline in these countries. While the existence of 120 schools with medical programmes in Mali has been reported as an asset to promoting health professions in rural areas [30], engagement of five Ugandan medical universities in 2010 to promote equitable health services for all the country’s population was documented to also stimulate rural medical practices in Uganda [43, 46]. In addition, in the DRC, Strasser et al. reported availability of rural health facilities to receive students for practical training as an important aspect to account for in the rural pipeline implementation.

Another reported facilitating factor of the rural pipeline is to ensure students’ accommodation and safety in rural areas. In the DRC, the Ministries of Health and Education proceeded in advance with the identification and preparation of specific rural sites that have the potential of accommodating an influx of students [29]. In Uganda, students recommended the availability of social amenities, affordable cost of living, their personal security in rural areas for their placement there [23].

In Mali, one strategy to promoting the implementation of the rural pipeline was to lift the age limit for candidates who were interested in attending training schools located in rural areas [31].

In South Africa and the DRC, having an appropriate academic capacity-building program for medical students has also been reported as a rural pipeline facilitator. This consists of setting, in rural medical schools, training programmes and supervision strategies that help students to successfully complete the courses and build their capacities such as caring for patients and engaging in teamwork [29, 44].

A set of motivational dynamics has been documented to be useful for implementing rural pipeline in Ghana and Mali. In Ghana, a partnership was built between a university located in the northern region (Tamale School of Medicine and Health Sciences) and community members to increase medical students’ awareness about the importance of working in rural areas [37]. In Mali, teachers from the Faculty of Medicine of Bamako encouraged students to settle in remote areas, sharing their positive experiences with them [26].

In Uganda, HWs said they would only work in rural settings upon active improvement of their wages [43]. As for them the important disparity between the salaries of HWs in urban areas and those in rural areas is a key consideration guiding their choice for their working places [43]. Non-financial incentives have also been reported in Mali as an encouraging strategy used to place health workers in rural areas [26]. These incentives included improving living (water, solar panels, motorbike) and working conditions (basic equipment, continuous education, peer support and mentoring) for young health professionals who accept to settle in rural Mali [26].

Studies from South Africa, Uganda and Ghana mentioned that students’ personal competencies for and interests in rural practice are also key factor to implementing rural pipeline [23, 38, 44]. Referring to competencies, students’ previous exposure to rural health facilities has been reported as a strong driver to working and getting committed to work at rural health facilities in Ghana and South Africa [38, 44]. Another reported asset was students’ ability to communicate in patients’ local language and their familiarity with local culture [23, 44]. As for students’ interests, these include a good on-site hospitality, the opportunity to spend time with their primary or extended families, the opportunity to explore rural areas and different clinical experience, and the opportunity for career advancement [24, 44].

In the particular context of Mali, marked by the overload of labour market in 1989, the government stopped recruiting HWs in urban areas [25]. This led young medical doctors facing unemployment challenges, to settle down in rural areas, with the encouragement of their university teachers and a Health NGO [25].

### Barriers of the implementation of rural pipeline

Barriers to the implementation of the rural pipeline were documented by seven studies in this review [23, 28, 30, 31, 38, 42, 46]. Seven main barriers were identified to hamper implementation of the rural pipeline in sub-Saharan Africa. They include poor preparation of rural medical school candidates, tuition fees, scarcity of medical schools in rural areas, limited access to rural health facilities, inadequate rural living conditions, perceived inappropriate working conditions in rural health facilities, and perceived discrimination of rural HWs.

Poor preparation of rural health training schools’ candidates has been found to decrease likelihood for rural candidates to be selected for medical studies, as these candidates studied in inappropriate secondary schools [30, 42]. Indeed, studies from Tanzania, South Africa and Uganda reported that most rural secondary schools not only lack science teachers to prepare them for admission in medical schools [42], but also are ill-equipped in terms of laboratories, computers, Internet and libraries [24, 30, 42].

A study in Mali reported that tuition fees and complementary educational costs can represent a barrier to accessing medical school, especially for rural students [31]. According to this study, the tuition fees varied in 2017 from 320 000 in public schools to 450 000 CFA (1USD = 500 CFA) in private schools, excluding living expenses, which is more difficult for rural inhabitants to bear [31]. As a consequence, the majority of medical school candidates come from cities (urban areas) [31].

One of the barriers to implementing rural pipeline in sub-Saharan Africa is the scarcity of medical schools in rural areas. Amalba et al. reported in 2019 that Ghana’s medical schools are mainly concentrated in the cities and that the country faces challenges to extend medical care to smaller towns and rural areas [38]. Kapanda et al. mentioned similar reasons hampering establishment of medical schools in rural Tanzania, that is, poor rural infrastructures (lack of electricity, poor roads, poor communication services) and general poverty of rural populations [42].

Limited access to health facilities in rural areas has been said to harden rural medical practices in Mali and South Africa [30, 31]. In Mali, health facilities that are selected to receive students are generally the easy-to-reach ones [31]. As such, the long distance, coupled with the poor transport and inadequate roads affect mobility of students or HWs to reach rural health facilities as testified by graduates in South Africa [30].

Studies in Mali and Uganda have pointed out the inadequate living conditions of medical students and HWs in rural areas, as a key factor making the latter ones unwilling to work in rural areas [23, 31]. These conditions mainly include the lack of support for subsistence and appropriate accommodation. In Mali for instance, students from private school have to pay the travel cost and other livelihood expenses themselves in rural areas for their internship [31]. In Uganda, the lack of appropriate accommodation for married HWs, and tutor for young medical students has been reported to be key obstacles for maintaining health professionals in rural areas [24].

Perceived inappropriate working conditions in rural health facilities have been documented as a barrier to achieving the rural pipeline in South Africa, Malawi, Uganda and Ghana. Medical students and health workers have reported across these countries that health professionals working in rural areas are commonly exposed to demotivating working conditions such as exceeding workload, lack of time for holydays, poor health equipment and infrastructures and poor support supervision from the hierarchy [24, 28, 30, 46]. These conditions were said to affect HWs’ job satisfaction and well-being [24, 30].

Atuyambe et al. reported two aspects related to the discrimination of rural HWs, as reasons for low retention of medical graduates in rural areas [46]. The first one is the perceived limited opportunity for career progression, such as specialization or short-term courses for skills and competence updates, especially for long-term rural practice. The second one is the poorer remuneration of rural health workers as compared to urban HWs.

## Discussion

This review contributes to complementing the existing literature regarding rural pipeline programmes implementation and their effects on rural health systems and communities in SSA. More specifically, it confirms the recommendations of updated WHO guideline on health workforce development, attraction, recruitment and retention in rural and remote areas from 2021 [4]. This scoping review indicates that a rural pipeline approach, as part of a bundle of broader interventions, contributes to retention of HWs in the SSA region. The scoping approach of the study did not allow assessment of the strength of the evidence for each rural pipeline pillar and strategy, hence now weight can be given to each of the recommendations below, in contrary to the WHO guidelines [4].

**Fig. 2:**
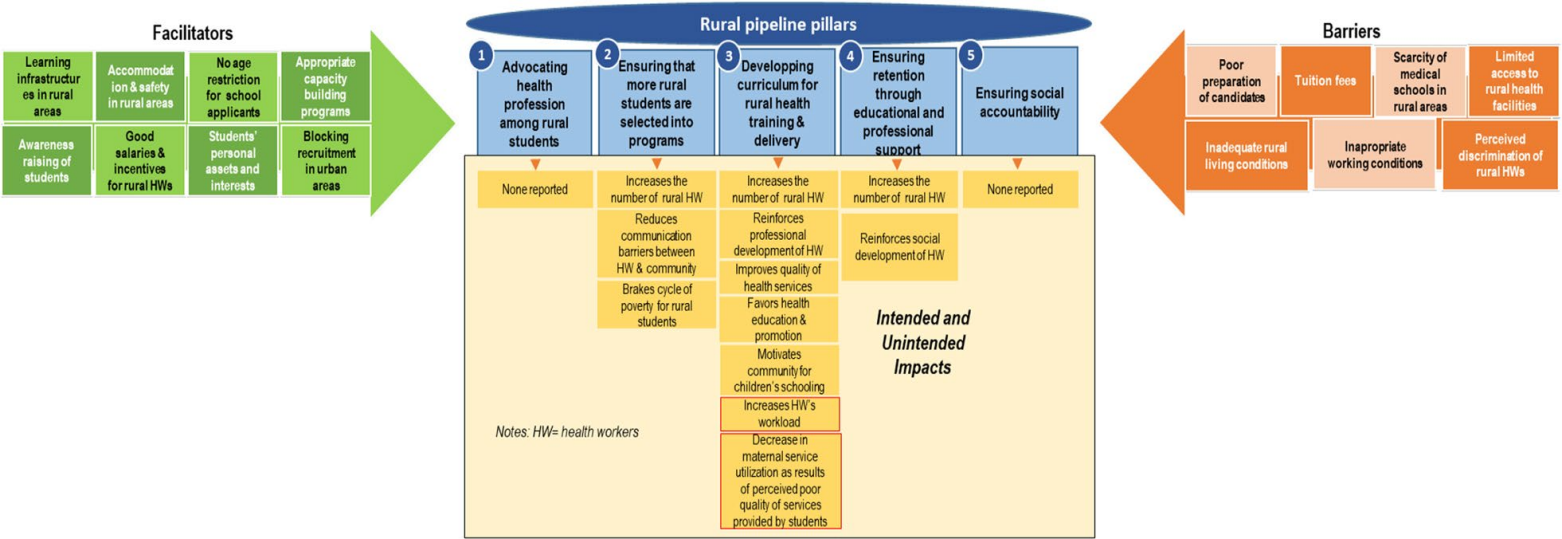
Conceptual framework of rural pipeline programmes implementation in sub-Saharan Africa

Rural pipeline strategies were reported to increase the number of rural health workers; to favour socio-economic well-being of health workers; to improve the quality of health services and access of rural community to healthcare; to reduce patient-provider communication barriers; to promote health education and promotion within rural communities; and to motivate communities to enrol their children in schools. Reviews on rural pipeline programmes have already been reported [51–53]. Holst et al. and Ogden et al. have shown that rural pipeline programmes have the potential to favour medical practice in rural and remote areas [51, 52]. Nevertheless, these reviews did not specify the potential of rural pipeline on improving the socio-economic development of health practitioners and rural communities access to quality health services. Another review conducted by MBemba et al. identified the effectiveness of rural pipeline programmes in improving health services utilizations [54]. However, this review did not stratify the effects of rural pipeline per pillar or strategy. Moreover, this review did not include studies from SSA. Our review fills in this gap by reporting on the effectiveness of the rural pipeline per pillar (Fig. 2).

**Fig. 3:**
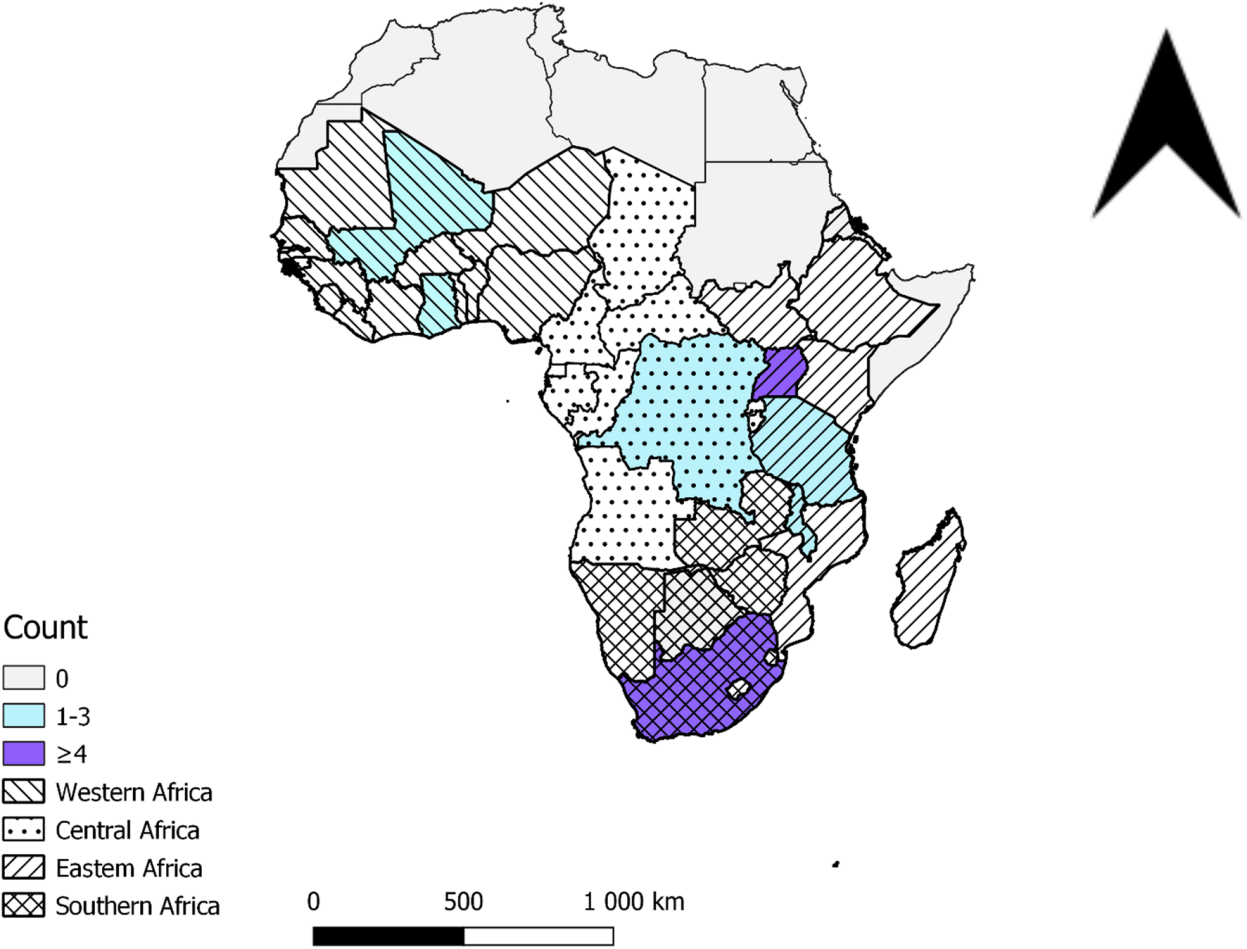
Distribution of rural pipeline studies aiming to increase the availability of health professionals in rural sub-Saharan Africa, 2007–2021

This review pulls out the key implications for policy actions necessary to implementing rural pipeline programmes namely, ensuring that more rural students are selected into programs; developing curriculum for rural health training and delivery; and ensuring retention through educational and professional support. Strategies that prioritize rural students in selection processes were identified to increase health practitioner intention and willingness to work in rural areas and to reduce communication barriers between patients and healthcare providers. This strategy also contributes to socio-economic development of rural communities through the breaking of poverty cycle for rural students. Strategies that ensure curriculum development for rural health training and delivery were acknowledged to increase the availability of health workers in rural areas, improve quality of health services, favour health education and promotion within rural communities, and motivate communities for children’s schooling. Strategies that ensure rural retention through educational and professional support were reported to increase the number of health professional in rural areas and reinforces their social development—learn from community culture and lifestyle, and benefit for their hospitality. Nevertheless, it is worth noting that these findings might not be generalizable to all SSA region, as most of the papers included in this review were published from Southern and Eastern countries of Africa (Fig. 3). This disparity is more evident between French (Western and Central regions) and English-speaking (Eastern and Southern regions) countries of Africa. In fact, only 4 out of 22 (18%) of the published studies included in this scoping review were from Francophone countries.

Our finding adds to the understanding of implementation challenges of complex interventions such as rural pipeline. First, we assessed that only few studies reported the combination of two and more components of rural pipeline [29–31, 36, 41, 49]. None of the studies included in this review combined all the four components of the rural pipeline intervention. In addition, our study shows that the adoption of this program is very rarely the consequence of a governmental reform; which result into their insular implementation by actors, depending on their interests, powers or resources (Table 2). Moreover, the implementation of all these components of the rural pipeline, however, requires significant funding and long-term political commitment. Whereas in the context of low-income countries, such programmes are often supported by external funding and are of short duration. Indeed, of the studies that have indicated the context that led to the pipeline programme reforms, almost all were funded by external mechanisms (Table 2). For example, authors in DRC and Uganda reported that funding for rural pipeline programmes was supported by external development funds, and over a period of 2–3 years [29, 43, 46]. In Mali, an international NGO was reported to support health professionals who settle in rural areas through the provision of better living conditions (water, solar panels, motorbike) and working conditions (basic equipment, continuous education, peer support and mentoring) [26]. Such external financial support can serve as a catalyst mechanism to sustain the implementation of health workforce development programmes. This means that, at local levels, prerequisites should exist in terms of infrastructures and equipment of health training institutions and health facilities, for such funding to contribute to an effective implementation of the health workforce development programme. Nevertheless, our study revealed a lack of adequate (primary and secondary) schooling and health facility infrastructures and equipment in some rural and underserved settings where pipeline programmes were implemented. Meeting such prerequisite would allow local recruitment and training of students into rural pipeline programmes, and favour future sustainable uptake of jobs in these locations. We also analysed that the achievement of rural pipeline programmes also relies on accompanying measures for the human resources at operational level such as students, local medical school lecturers, supervisors of students at health facility level. These measures may include appropriate housing; scholarship for students to remove the barrier of training costs but also for their subsistence; incentives for the rural supervision health workers; adequate wage for rural HWs and other amenities for good living conditions of the lecturers generally coming from cities such as electricity, roads to easily access cities, communication (phone network, internet), appropriate markets. These findings raise calls for the prioritization of interconnected interventions, tailored with specific country contexts and with full commitment of government and (intern) national stakeholders for investing in the health sector to address health workforce placement related challenges. It also entails encouraging inter-sectoral collaborations in the implementation of health workforce strategies for an improved feasibility (through the meeting of prerequisites such as appropriate living and working conditions for the health workforce) and sustainability of its impacts on local health systems and services.

**Table 2:** Studies undertaken under a context of health workforce development in sub-Sahara Africa

Countries	Authors’ name	Who undertook the Workforce development intervention? How long the intervention lasted?	In which context the intervention was undertaken?	What was achieved by the intervention?^a^
Mali	Sidibé et al. 2019	The Government of Mali undertook a decentralization of training schools in rural regions	This decentralization reform attempted to improve accessibility of care	• Revision of curricula to include rural health topics; • Expansion of training schools in rural regions; in 2017, 115 to 120 training schools existed in rural Mali; • Increased in number of graduates; from 259 to 340 annually for midwives and from 261 per year in 2013 to a thousand per year in 2017 for obstetric nurses; • 92% of graduates (mainly midwives) remained in the rural regions after their trainings
	Dormael et al. 2008	The NGO “Santé-Sud” and the Rural Doctors Association decided to set up an orientation course for recently established rural doctors	• Data suggest that community doctors face unforeseen situations for which they feel ill prepared, leading sometimes to early dropout (poor retention); • An orientation course on rural practice was developed for newly established rural doctors from 2003 to 2005 with the assumption that training meeting rural practitioners' needs would strengthen young doctors' technical competences and self-confidence, and consequently contribute to retention	• An orientation course on rural practice for newly established rural doctors from 2003 to 2005 contributed to a positive impact on their preparedness for rural practice and increased retention in rural areas, with 50% still in rural practice after 4 years; • In 2007, 99 rural doctors were serving in over 13% of Mali's rural community health centres
	Coulibaly et al. 2007	• The NGO “Santé-Sud” encouraged young doctors, facing unemployment, to settle in rural areas by providing them with installation kits (medicines, equipment, etc.), solar panels, a motorcycle, and a prerequisite training; • A regular follow-up is also organized as well as the support the professional association to help them fight against professional isolation: Regional and national meetings, continuing education, reciprocal visits, etc.	• Healthcare system and decentralization reforms in 1999; • The healthcare system reform of the 1999 resulted in the rise to new actors, the Town Halls, responsible for the Community Health Centres (CSCom). It is in this context (of reform of the health care system and decentralization) that rural medicine has developed in Mali; • Confronted with the banning of recruitment in urban areas, because of the healthcare system and decentralization reforms, many young doctors took up initiatives to settle in rural areas	• In 2005, 74% of young doctors supported by the ONG Santé-Sud were practising at CSCom level; • 11% of them had a contract with other types of associations (11%) or choose to practice in private practice (15%); • Data showed a relative stability of rural doctors: by 2005, 45% had been practising for more than 5 years, and 25% planned to practice for more than 10 years; • Many of those who left the profession became district chiefs, in charge among others to support CSCom
Ghana	Amalba et al. 2016a, 2016b, 2019	• International reforms in medical education (2007); • In response to the international changes in medical education, The University for Development studies (UDS), School of Medicine and Health Sciences introduced a Problem-Based Learning and Community-Based Education and Service (PBL/COBES) methodology in its medical training curricula, in replacement of the traditional medical training curricula	• The programme occurred in a context where the Ghanaian government was offering a 20–30% salary top-up and a staff vehicle hire purchase scheme for health staff working in rural areas	• 74% of students from PBL/COBES track indicated that their medical school curriculum adequately prepared them for rural practice as compared to those from the traditional track (35%); • Significant proportions of program’s graduates from the towns (61%) and cities (68%) indicated that the PBL/COBES programme influenced them to practice in rural area; •••••••••44% of the students indicated that PBL/COBES have had an influence on their choice of career specialty
South Africa	Gumede et al. 2021	• The Umthombo Youth Development Foundation (UYDF) undertook the intervention based on evidence from Australia and Canada; • The programme commenced in January 1999 in provinces of KwaZulu-Natal and the Eastern Cape, areas which lacked good health and education services, and where unemployment was high		• The UYDF addressed the shortage of healthcare workers in the provinces of KwaZulu-Natal and the Eastern Cape, by identifying young people who were eligible for scholarships in health sciences; • The programme commenced with four students and by the end of 2017 had produced 336 graduates and was supporting 251 students with an annual pass rate of over 90%
	Schalkwyk et al. 2018	• In the 1990s, the Faculty of Medicine and Health Sciences at Stellenbosch University initiated Community-based Education as a strategy to train students appropriately for delivering primary health care services to South African communities		• By 2015, 80 primary healthcare sites were covered by the short clinical rotations programme
	Mapukata et al. 2017	• The Faculty of Health Sciences at the University of Witwatersrand was the launch of the Graduate Entry Medical Programme in 2003 as a 4-year training programme that complemented the existing traditional approach to medical training, with both streams being combined in the clinical years • Within that, the integrated primary care (IPC) block, which is a compulsory 6-week placement in a range of primary health care settings, was launched in 2006 as one of the initiatives that would strengthen the university’s and students’ commitment to rural and underserved communities		
Uganda	Kizito et al. 2017	• In 2010, Five Ugandans medical universities formed a consortium, Medical Education for Equitable Services to All Ugandans (MESAU), in order to address challenges in health professions education • These universities are: Makerere University College of Health Sciences, Mbarara University of Science and Technology, Gulu University, Kampala International University and Busitema University • This 5-year programme was established with funding from the US Government through the Medical Education Partnership Initiative (MEPI) and technical support from Johns Hopkins University		• The MESAU institutions included Community-Based Education, Research and Service (COBERS) in their health professions curricula; • Before COBERS, 44% students indicated that they intended to work in rural areas as compared to 48% after the COBERS placement • Before the COBERS placement, the factors that were associated with students considering to work in a rural area were: extra allowance (OR = 0.2; 95% CI 0.1–0.6), and availability of social amenities (OR = 0.2; 95% CI 0.1–0.7). After their COBERS placement, the factors were: access to long distance courses (OR = 2.0; 95% CI 1.0–3.7) and being posted to a facility in a rural area (OR = 15.0; 95% CI 6.5–35.5); • Before the COBERS placement the factors that influenced how long students thought they would be willing to work in a rural environment were: reliable electricity (IRR = 0.6; 95% CI 0.3–1.0) and Internet (IRR = 1.5; 95% CI 1.0–2.3), high salary (IRR = 0.4; 95% CI 0.3–0.7), and having skills to practice in rural settings (IRR = 2.0; 95% CI 1.3–3.1). Reliable electricity (IRR = 0.5; 95% CI 0.3–0.8) and long distance courses (IRR = 2.1; 95% CI 1.4–3.1) were significant motivators after having undergone the COBERS placement
	Atuyambe et al. 2016	• MESAU is the first nation-wide consortium approach to addressing medical education in Uganda with the overall aim of standardizing medical education and developing the partner institutions as centres of excellence for medical education, research and service that ad dress local and national needs to improve health in Uganda; • One of MESAU’s objectives is to improve the quality and relevance of medical education in order to produce health workers with the competencies and motivation to deliver locally relevant services; • Each of the MESAU institutions has implemented CBE as an integral part of their respective curricula for varying lengths of time since 1989		
	Kaye et al. 2010	• The Makerere University Faculty of Medicine changed the traditional training of medicine and nursing students to a problem-based learning (PBL) curriculum in 2003; • The first of the nursing and medicine PBL graduates completed their studies in 2007 and 2008, respectively; • A comprehensive evaluation of the PBL curriculum aimed to assess the influence of graduates’ training on their choice of workplace		
DR Congo	Strasser et al. 2021	• With PEPFAR funding through the United States Health Resources and Services Agency (HRSA), in 2017, ICAP was awarded the Resilient and Responsive Health Systems (RRHS)		• The first 2 years of the RRHS project built on extensive HRH capacity-building and infrastructure improvements for student nurses and midwives through the HRSA-funded Nursing Education Partnership Initiative and Global Nursing Capacity Building Programme
Malawi	Kelly et al. 2015	• In 2004, the Malawi Ministry of Health (MOH) and the Christian Health Association of Malawi (CHAM) began a 6-year Emergency Human Resource Programme (EHRP) to address the critical health worker shortage; • The Global AIDS Interfaith Alliance (GAIA), a non-profit organization, also attempted to address the nursing shortage in Malawi • GAIA offers preservice scholarships for nurses who need assistance with college fees and who demonstrate a commitment to work in the public health system after graduation; • The scholarship program supports nursing students who are predominantly orphans or from lower socio-economic backgrounds		• The program has been operating in Malawi in collaboration with the MOH, CHAM, and the training institutions since 2005 and has awarded more than 400 scholarships to nursing students at the technician, diploma, bachelor’s, and master’s level; • The MOH also deploys 40% of nurse graduates to CHAM facilities while the other 60% are deployed to MOH facilities

^a^More detail in Table 1 (column on rural pipeline outcomes)

Another finding that calls for policy considerations is the limited attention given to the allied health professions (nurses, midwives) in the implementation of the rural pipeline. Only five of the articles included in this study reported on rural pipeline programmes targeting nursing and midwifery learners [23, 24, 28, 29, 31]. However, this result contrasts with the socio-economic realities of low-income countries such as those in SSA. Indeed, medical training requires more time, human resources (teachers) and financial resources (training and equipment costs) than nursing and midwifery trainings. Also, retaining medical doctors in rural areas appears to be more difficult and costly than other professional categories [55–62]. However, nurses and midwives constitute the bulk of maternal and child health care providers [63]. Therefore, investing in the development of auxiliary health workers such as nurses and midwives is likely cost-effective in the African context and would lead to faster results in improving health workforce availability and health indicators rural communities.

This review has identified an additional pillar to the already documented pillars [10]. This relates to social accountability; that is ensuring that students will meet community health needs after graduation. It builds on strategies such as matching students’ socio-demographic profiles to the communities and ensuring they remain in communities they are assigned to, after their placement. We consider this pillar a key asset of the rural pipeline programme as it is a favourable outcome based on the other pillars such as promoting rural recruitment of students, supporting their training through scholarships, creating adequate conditions for training and internship in rural areas, exposing them to the rural realities of care during training.

## Limitations

The present review has some limitations. First, the methodological design of this study—scoping review—did not allow any quality assessment of included studies. Moreover, given that only two researchers checked and agreed on the database, there might be a risk of selection bias. This could limit the internal validity of the effectiveness of rural pipeline interventions reported in the present study. A meta-analysis study design could overcome this but given the criteria needed for inclusion in such a review, it is likely that very few articles would be included. Second, the geographical disparity of selected studies, with most studies published from Eastern and Southern regions could undermine the generalization of findings to the whole SSA. This could also hamper the external validity of the findings to other low-income countries. We argue for future studies to include an explorative case-study design for specific countries as this would provide for more contextual and robust evidence on the effectiveness, and eventual impact, of the rural pipeline implementation.

## Conclusion

This scoping review shows that rural pipeline programmes potentially impact rural health systems and services by increasing the number of rural health practitioners; reducing communication barriers between healthcare providers and community members; changing household economic and social circumstances especially for students from poor family; and improving the quality of health services. However, rural pipeline can result in some unintended impacts such as perceived workload increased by trainee’s supervisors; increased job absenteeism among senior health providers; discomfort of being attended by students; and perceived poor quality care provided by students. Additionally, poor preparation of rural health training schools’ candidates; tuition fees payment; limited access to rural health facilities for students training; inadequate living and working conditions; and perceived discrimination of rural health workers hamper the effective implementation of rural pipeline approach in sub-Saharan Africa. We recommend interconnecting interventions and tailoring them with country specific contexts to address rural health workforce development related challenges hereby improving rural pipeline programmes implementation and their impact in rural sub-Saharan Africa. Moreover, there is a need to undertake more health workforce development interventions targeting nursing and midwifery professions in Sub-Saharan Africa. Finally, we call for more governmental commitment for improving rural living, and working environments to facilitate the implementation of rural health workforce development programmes.

## Declarations

### Availability of data and materials

All materials used in this study may be available upon request to LC at: codjial@who.int.
